# Emergence and Prevalence of an African Swine Fever Virus Variant in Wild Boar Populations in South Korea from 2019 to 2022

**DOI:** 10.3390/v15081667

**Published:** 2023-07-31

**Authors:** Garam Kim, So-Jeong Kim, Won-Jun Kim, Jung-Hyeuk Kim, Ji-Chul Kim, Sang-Geon Lee, Eun-Sol Kim, Sang-Hyun Lee, Weon-Hwa Jheong

**Affiliations:** Wildlife Disease Response Team, National Institute of Wildlife Disease Control and Prevention (NIWDC), 1 Songam-gil, Gwangsan-gu, Gwangju 62407, Republic of Korea; garam1204@korea.kr (G.K.); th3555@korea.kr (S.-J.K.); kwj22081@korea.kr (W.-J.K.); rlawjdgur01@naver.com (J.-H.K.); chulkim@korea.kr (J.-C.K.); rmsidrnt@icloud.com (S.-G.L.); dnjsdmsthf@naver.com (E.-S.K.); ishmael6410@korea.kr (S.-H.L.)

**Keywords:** African swine fever virus, genome, multigene family, next-generation sequencing, central variable region

## Abstract

African swine fever (ASF), a viral disease caused by the African swine fever virus (ASFV), is associated with high mortality rates in domestic pigs and wild boars. ASF has been spreading since its discovery in wild boars in Korea in October 2019. Genomic analyses have provided insights into the genetic diversity of the ASFV isolated from various regions, enabling a better understanding of the virus origin and transmission patterns. We conducted a genome analysis to evaluate the diversity and mutations of ASFV spreading among wild boars in Korea during 2019–2022. We compared the genomes of ASFV strains isolated from Korean wild boars and publicly available ASFV genomes. Genomic analysis revealed several single-nucleotide polymorphisms within multigene families (MGFs) 360-1La and 360-4L in Korean ASFV. MGF 360-1La and 360-4L variations were not observed in other ASFV strains, including those of genotype II. Finally, we partially analyzed MGFs 360-1La and 360-4L in ASFV-positive samples between 2019 and 2022, confirming the geographical distribution of the variants. Our findings can help identify new genetic markers for epidemiological ASFV analysis and provide essential information for effective disease management.

## 1. Introduction

African swine fever (ASF), a highly contagious hemorrhagic disease caused by the African swine fever virus (ASFV), is a major cause of death in domestic pigs and wild boars [1]. ASF transmission can occur through direct contact with infected animals or indirect contact with contaminated materials such as equipment, clothing, feed, and ticks [2]. ASF was first identified in Kenya in 1921, which later spread to Africa, Europe, and Asia. Furthermore, ASF was also reported in the Dominican Republic and Haiti [3,4,5,6]. In Korea, ASF has spread nationwide since the first case in wild boars in October 2019 [7]. Therefore, the Korean government has been implementing measures, including fence cutting, population control, carcass searching, and safe disposal, to control the spread of ASF in domestic wild boars [8,9]. Despite these efforts, new cases of ASF occurring in wild boars are still being reported in Korea.

ASFV is a member of the Asfarviridae family and is a large icosahedral DNA virus [10]. The ASFV 170–190 kb genome encodes 150–170 open reading frames (ORFs) [11]. Most ORFs encode structural and nonstructural viral proteins involved in viral replication, transcription, translation, and virulence [12]. Demand for the complete ASFV genome sequence has surged owing to the rapid spread of the pathogen [13]. Moreover, the virus is an emerging threat to the swine industry; hence, detailed genetic information is urgently required to understand its biology [14]. Therefore, studies are being conducted to better understand the genetic diversity, evolution, and virulence of different ASFV strains [15].

The ASFV genomic diversity has been explored using pairwise sequence similarity and ORF distribution comparisons [16]. The nucleotide sequences of ASFV genomes have been compared to determine their similarity, and the genetic variations in ASFV and factors influencing its evolution have been revealed [11]. ASFV strains are classified into 24 genotypes based on the nucleotide sequence analysis of the *B646L* (*p72*) variable region, with all gene variants associated with the disease [17,18]. Over 140 ASFV genome sequences are available from various sources, exhibiting varying virulence degrees from different geographical locations. Sequence analysis of variable genomic regions is extensively used in molecular ASFV epidemiological studies [16]. *X64R*, *EP152R*, *EP153R*, *EP402R*, *EP364R*, and *CP2475L* have been identified in regions characterized by a high genetic ASFV diversity [19]. High variability is observed within the 35 kb at the 3′ end and the 15 kb at the 5′ end of the genome [20,21]. These two regions contain the multigene families (MGFs); ASFV has five MGFs, namely MGF-100, -110, -300, -360, and -505 [22]. The ASFV genome MGF region is highly variable and characterized by frequent mutations and genetic rearrangements [23]. Genes within the ASFV MGF-360 and MGF-505 regions reportedly inhibit host interferon production and are associated with host specificity, innate immune mechanisms, and virulence [24].

Analyzing ASFV isolates from Poland using whole-genome sequencing has revealed single-nucleotide polymorphisms (SNPs). These SNPs were identified in the *K145R* and *MGF 505-5R* genes, distinguishing the Polish isolates from the reference Georgia 2007/1 strain [25]. During the initial ASF outbreak in India, complete genome sequencing revealed several distinct nonsynonymous mutations in *MGF 369-11L* and *505-4R* [26]. In addition, recent epidemiological simulations of the spread of the ASFV variant and its genetics have demonstrated the emergence of probabilistic and geographically distinct variant clusters in nonselective populations of wild boars. Furthermore, specific mutation sites have been identified as genetic markers, enabling genome dynamics analysis of various ASFV outbreak variants in Germany [27]. Therefore, identifying intriguing genetic variations in Korean wild boar ASFV is essential, as it will help distinguish isolates from Korea and those from other regions, and provide valuable information for specific diagnosis and treatment opportunities.

In this study, we aimed to analyze the ASFV genome isolated from ASFV-infected wild boars in Korea during 2019–2022. We identified characteristic mutations in the MGF 360-1La and MGF 360-4L regions for the first time. These mutations exhibited geographically distinguishable cluster patterns. The findings of this study, which reveal the genetic diversity of ASFV in domestic wild boars, have major implications. The mutations can serve as valuable genetic markers for conducting epidemiological analyses and developing preventive measures. Furthermore, the comprehensive analysis of ASFV diversity based on its distinct characteristics within the country can provide essential data for the development of effective vaccines.

## 2. Materials and Methods

### 2.1. Analysis of ASF Outbreak Distribution

We obtained data on ASF diagnosis in Korean wild boars, and these results were deposited in the World Organization for Animal Health. We analyzed ASF occurrences during 2019–2022 and used open-source Geographic Information System software version 3.24 (www.qgis.org, accessed on 25 July 2023) to investigate the geographical distribution of these outbreaks. A cartographic analysis was conducted based on the geographical coordinates of each ASF outbreak.

### 2.2. DNA Extraction and ASFV Detection in Wild Boar Samples

The Maxwell RSC Viral Total Nucleic Purification Kit (Promega, Madison, WI, USA) was used to extract DNA from the blood of wild boars, according to the manufacturer’s instructions. Polymerase chain reaction (PCR) was performed to detect the presence of ASFV DNA, using the ASFV diagnostic primers *PPA1* (5′-AGTTATGGGAAACCCGACCC-3′), *PPA2* (5′-CCCTGAATCGGAGCATCCT-3′) [28], *P72D* (5′-GTACTGTAACGCAGCACAG-3′), and *P72U* (5′-GGCACAAGTTCGGACATGT-3′), which partially amplified *B646L* (p72) [17]. The MGF 360-1La and 360-4L regions of ASFV were detected using the primers MGF 360-1La (F: CCGATTAATGTCAGCCCCCA, R: TGCAGACATCAGCTTTGGGT) and MGF 360-4L (F: CTCTAAGGCACGGTCAAGGT, R: TTCCTCATTTAGATCTTTGGCGT). The cycling protocol comprised an initial step at 95 °C for 2 min, followed by 45 denaturation cycles at 95 °C for 20 s, annealing at 58 °C for 40 s, and extension at 72 °C for 40 s. After the cycling steps, a final extension was performed at 72 °C for 5 min. Electrophoresis was performed using 1.5% agarose gels to visualize the amplification products. The gels were stained with Ecodye (BIOFACT, Seoul, Republic of Korea). Sanger sequencing was performed by Macrogen Inc., an outsourcing service specializing in Sanger sequencing, to determine the nucleotide sequence of the PCR products.

### 2.3. Complete ASFV Genome Sequencing

The Maxwell Viral Total Nucleic Purification Kit (Promega) was used to extract total DNA from 200 μL whole blood from wild boars, following the manufacturer’s instructions. As outlined in the OIE manual, the samples were amplified through PCR using the PPA1/PPA2 and P72 primers to detect ASFV. Library preparation was performed using an enzymatic preparation kit (Celemics, Seoul, Republic of Korea) after shearing genomic DNA (gDNA). Subsequently, the gDNA library and capture probes were hybridized using a target enrichment kit (Celemics), which includes chemically synthesized capture probes hybridized to the target regions. Post-PCR amplification was performed to enrich the captured regions. The target-captured library was sequenced against a next-generation sequencing library that was generated using the Illumina NextSeq 550 platform (Illumina, San Diego, CA, USA), with a target capture 2 × 150 bp read layout. Adaptor sequences and low-quality bases were trimmed using the Fastx Toolkit 0.0.14 (http://hannolab.cshl.edu/fastx_toolkit/, accessed on 25 July 2023), and AdapterRemoval (version 2.2.2) was used to trim the sequences. The reads were mapped onto a reference ASFV genome (accession number: FR682468) using Burrows–Wheeler Aligner software version 0.7.10. SNPs, indels, and structural variation were detected using Genome Analysis TK 4.0.4.0. The read alignment quality was assessed using the SAMtools software (samtools 1.1) and Python software package (numpy 1.11.0).

### 2.4. ASFV Complete-Genome Annotation

We used Genome Annotation Transfer Utility (GATU) software available at the Viral Bioinformatics Resource Center to annotate the newly constructed genome. We used the Georgia2007 strain as a reference for annotation.

### 2.5. Genetic ASFV Characterization and B646L (p72) Phylogenetic Analysis

We selected 16 closely related viruses and constructed a maximum likelihood (ML) phylogenetic tree of their whole-genome sequences to explore the phylogenetic relationships among different ASFV strains. We used Randomized Axelerated Maximum Likelihood version 8.0 with default parameters and a general time-reversible model, accounting for the gamma-distributed rate variation among sites. The genome sequences were aligned using multiple alignment within a fast Fourier transform algorithm in Geneious Prime version 2023.0.4 (https:www.geneious.com/prime, accessed on 25 July 2023). We constructed an ML phylogenetic tree of *p72* using Molecular Evolutionary Genetics Analysis (MEGA) X to assess support for our phylogenetic tree. We performed 1000 replicates of ML bootstrapping to evaluate the robustness of the tree topologies. In addition, we aligned the nucleotide sequence of *B646L* (*p72*) of the Korean ASFV strains with those of other ASFV strains belonging to the same *B646L* (*p72*) genotype using the ClustalW algorithm in MEGA X. The evolutionary history was inferred using the maximum composite likelihood model method. We used the neighbor-joining method with 1000 bootstrap replicates to construct the phylogenetic tree.

## 3. Results

### 3.1. Geographical Distribution of ASF Outbreaks in South Korea during 2019–2022

**Figure 1:**
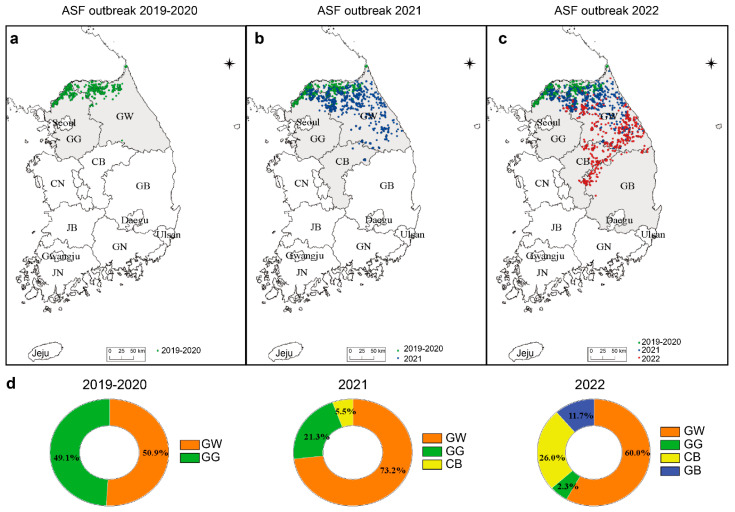
Analyzing the territorial distribution of African swine fever (ASF) throughout Korea during 2019–2022. The wild boar ASF outbreak is shown on the map by year, with (**a**) green dots representing 2019–2020, (**b**) blue dots for 2021, and (**c**) red dots for 2022. (**d**) ASF regional outbreaks between 2019 and 2022: orange, Gangwon-do; green, Gyeonggi-do; yellow, Chungcheongbuk-do; blue, Gyeongsangbuk-do. GW, Gangwon-do; GG, Gyeonggi-do; CB, Chungcheongbuk-do; CN, Chungcheongnam-do; GB, Gyeongsangbuk-do; GN, Gyeongsangnam-do; JB, Jeollabuk-do; JN, Jeollanam-do.

The ASF outbreaks in South Korea during 2019–2022 are shown in Figure 1. The first outbreak occurred in Yeoncheon (Gyeonggi-do) in October 2019, followed by Yeoncheon (Paju) and Cheorwon (Gangwon-do). In 2020, the ASF epidemic spread from the western to the eastern regions. Of the total ASF cases occurring during 2019–2020, 49% and 51% occurred in Gyeonggi-do and Gangwon-do, respectively (Figure 1a,d). In 2021, the ASF cases reported in Gyeonggi-do, Gangwon-do, and Chungcheongbuk-do accounted for 21%, 73%, and 5% of the total cases, respectively. Compared with that during 2019–2020, the ASF incidence rate in Gyeonggi-do decreased in 2021 (Figure 1b,d). While the incidence rate in Gangwon-do decreased in 2022 compared with that in 2021, it remained the highest between 2019 and 2022, particularly in the southern region. In Gyeonggi-do, which had an incidence rate similar to that in Gangwon-do during 2019–2020, the incidence decreased between 2021 and 2022 to 2.3% of the total incidence rate. After the first occurrence in 2021 in the Chungcheongbuk-do region, the incidence rate of ASF substantially increased 4.7 times in 2022, and a new outbreak occurred in Gyeongsangbuk-do (Figure 1c,d). These results show that the occurrence diverged southward from the northernmost region of Korea.

### 3.2. Geographical Representation of the ASFV Genome Isolated from a Wild Boar with ASF in South Korea

**Figure 2:**
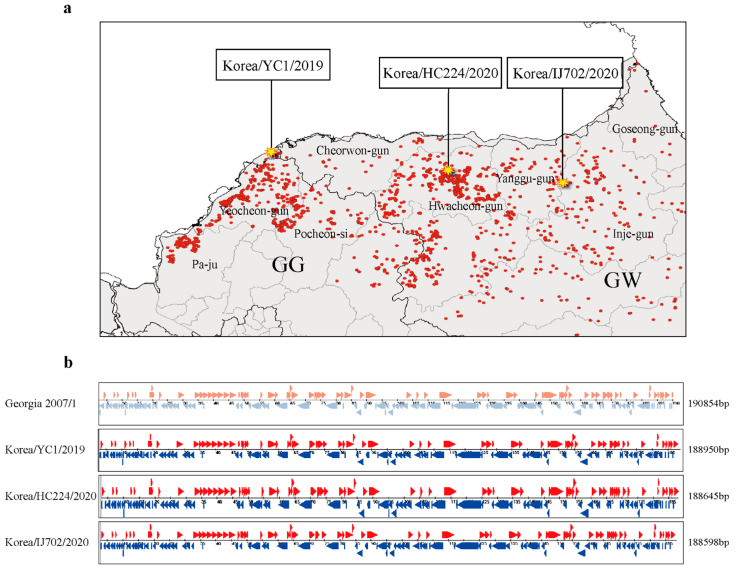
The genome of the African swine fever virus (ASFV) obtained from a wild boar in South Korea was mapped geographically. The red dots on the map indicate the occurrence of ASF in wild boars from 2019 to 2022 (**a**) From left to right, the asterisks indicate the areas of Yeoncheon, Hwacheon, and Inje from where the ASFV genome has been registered in NCBI. (**b**) Visualization of the open reading frames (ORFs) of ASFV in Korea/YC1/2019, Korea/HCC224/2020, and Korea/IJ702/2020.

We generated the whole-genome sequences of ASFV isolated from wild boars with ASF in Hwacheon and Inje in 2020 during the active spread of the outbreaks (Figure 2a). The complete genome sequences of the ASFV Korea/HC224/2020 and Korea/IJ702/2020 strains, with total lengths of 188,645 bp and 188,598 bp, respectively, were determined; the GC content was 38.4%. Korea/HC224/2020 and Korea/IJ702/2020 strain sequence annotations were created using the GATU software [29]. The genome length of Georgia 2007/1 is 190,854 bp, and sequence differences are present in the 5′- and 3′-ITR regions [30], resulting in an approximate 2000 bp difference compared to that in the YC1/2019, HC224/2020, and IJ702/2020 strains (Figure 2b).

In the Korea/HC224/2020 and Korea/IJ702/2020 strains, 46 MGF genes were identified, namely, MGF-100 (three members), -110 (11 members), -300 (three members), -360 (19 members), and -505 (10 members). The Korea/HC224/2020 strain genome sequences have been deposited in GenBank under accession number OP628183.

### 3.3. Phylogenetic Analysis of Complete ASFV Strain Genomes

**Figure 3:**
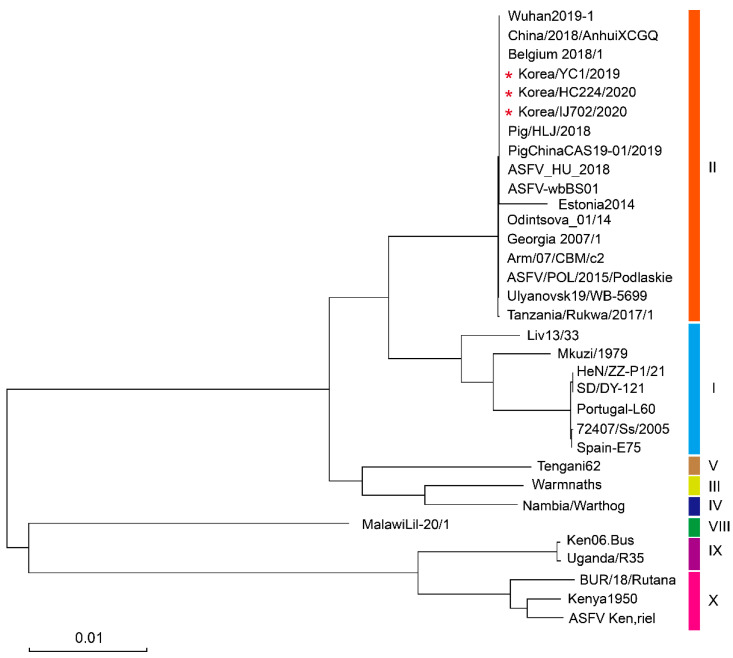
Maximum likelihood phylogenetic tree constructed from ASFV genome sequences. The scale bar represents the number of substitutions per site, and the node values are shown as bootstrap values (1000 replications). The red asterisk represents the ASFV strain that occurred in wild boars in South Korea.

A phylogenetic tree of ASFV *p72* was constructed to determine the genetic relationship between HC224/2020 and IJ702/2020 strains and the other isolated ASFV strains. An ML phylogenetic ASFV dendrogram was constructed by aligning genome sequences. A phylogenetic analysis of the ASFV genome showed that the strains clustered according to genotype. The HC224/2020 and IJ702/2020 strains belonged to *p72* genotype II (Figure 3).

### 3.4. ASFV Genome Comparison of Korean Strains with Reported ASFV Strains

**Figure 4:**
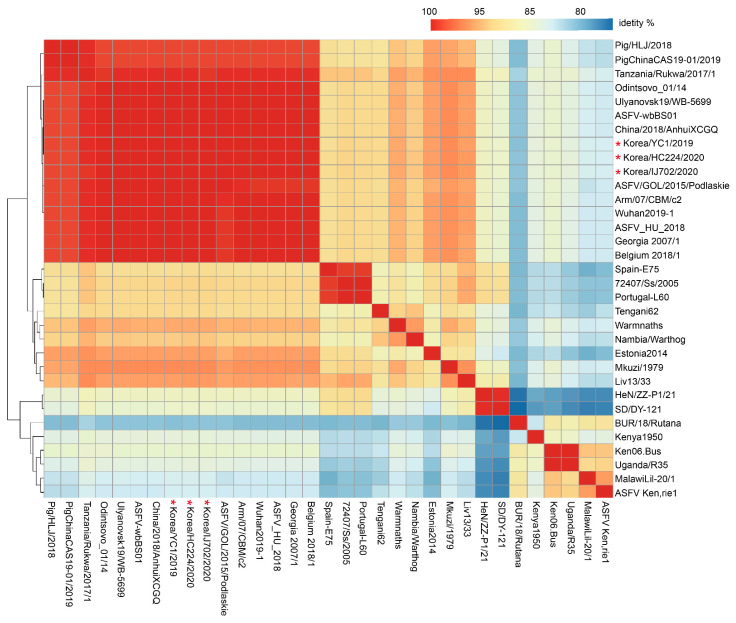
The sequence similarity matrix plot displays pairwise comparisons of ASFV genome sequences in the curated dataset. The similarity degree between genome sequence pairs is represented by different colors, with dark red indicating 100% identity and blue indicating lower identity. The red asterisk represents the ASFV strain that occurred in wild boars in South Korea.

We compared pairwise sequence similarities to investigate the genome-wide gene content of 30 ASFV strains. Table 1 shows the strain information, and the ML phylogenetic tree of the ASFV genome sequences is shown on the left (Figure 4).

The overall whole-genome sequence similarity varied between 75.37% and 99.9%. The sequence divergence observed within genotype II was wide, varying between 91.42% and 99.99%. Similarly, the pairwise sequence similarities observed among Korean ASFV strains were highly divergent within genotype I, with a range of 86.5%–90.8%. The highest inter-genotype pairwise similarity was observed for genotype II (Georgia 2007/1, ASFV_HU_2018, China/2018/AnhuiXCGQ, Belgium 2018/1) at 99.9%. In contrast, the lowest inter-genotype pairwise similarity was found between genotypes X (Kenya1950, Ken.rie1 and BUR/18/Rutana) and VIII (MalawiLil-20/1), at 80.54% and 85.39%, respectively. Although the Korea/HC224/2020 and Korea/YC1/2019 strains showed 100% sequence similarity, the Korea/IJ702/2020 strain exhibited 99.97% similarity to the Korea/HC224/2020 and Korea/YC1/2019 strains.

### 3.5. Amino Acid Sequence Alignment in the MGF 360-1La and 360-4L Regions

We analyzed similarities in the genomes of Korea/HC224/2020, Korea/YC1/2019, and Korea/IJ702/2020 strains, and found three novel mutation sites (MGFs 360-1La, 4L, and 360-10L) when comparing the Korea/HC224/2020 and Korea/IJ702/2020 strains with the Korea/YC1/2019 strain. Two synonymous mutations (MGFs 360-1La and 360-4L) and one nonsynonymous mutation (MGF 360-10L) were observed (Table 2).

**Figure 5:**
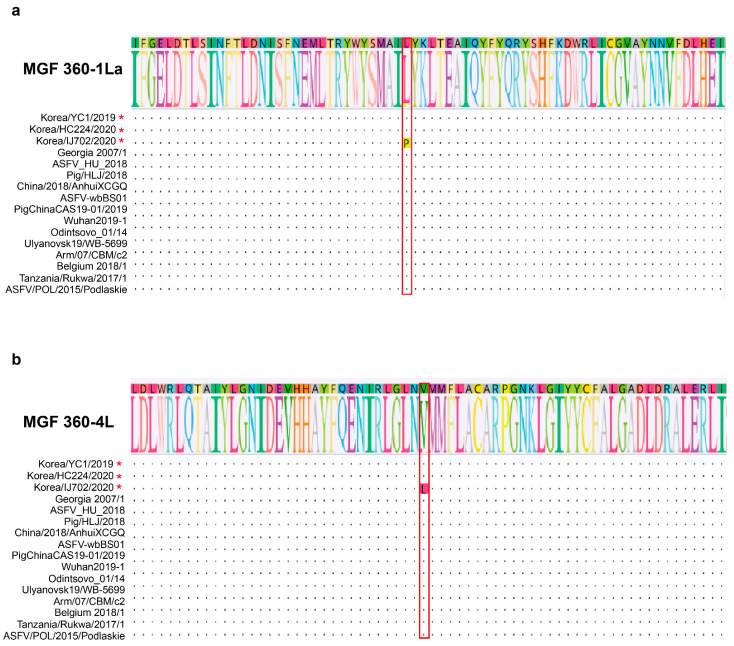
Comparison of amino acid sequences in the MGFs 360-1La and 360-4L regions between the Korean wild boar ASFV strain and other ASFV strains. The partial amino acid sequence of the MGF-360 1La (**a**) and MGF-360 4L (**b**) regions of ASFV was aligned. Each character represents a single amino acid according to the international nomenclature. During amino acid alignment, identical amino acids were marked with dots, and isolates highlighted in different colors belong to different groups based on sequence variations caused by mutations. The red asterisk represents the ASFV strain that occurred in wild boars in South Korea.

MGFs 360-1La and 360-4L are encoded in the reverse strand sequence of the left variable region of the ASF genome. MGF 360-1L is upstream of MGF 360-2L, and MGF 360-4L is between MGFs 110-13L and 360-6L. MGFs 360-1La and 360-4L encode proteins comprising 277 and 387 amino acids, respectively. The sequence analysis revealed an SNP within MGFs 360-1La and 360-4L. The amino acid at MGF 360-1La position 106, leucine (L), is replaced with proline (P). In MGF 360-4L, the amino acid at position 243, valine (V), is replaced with leucine (L) (Figure 5a,b). SNPs were identified using the data from aligning the sequence to the Georgia 2007/1 strain.

### 3.6. Geographic Distribution of Variant ASFV in Korea during 2019–2022

**Figure 6:**
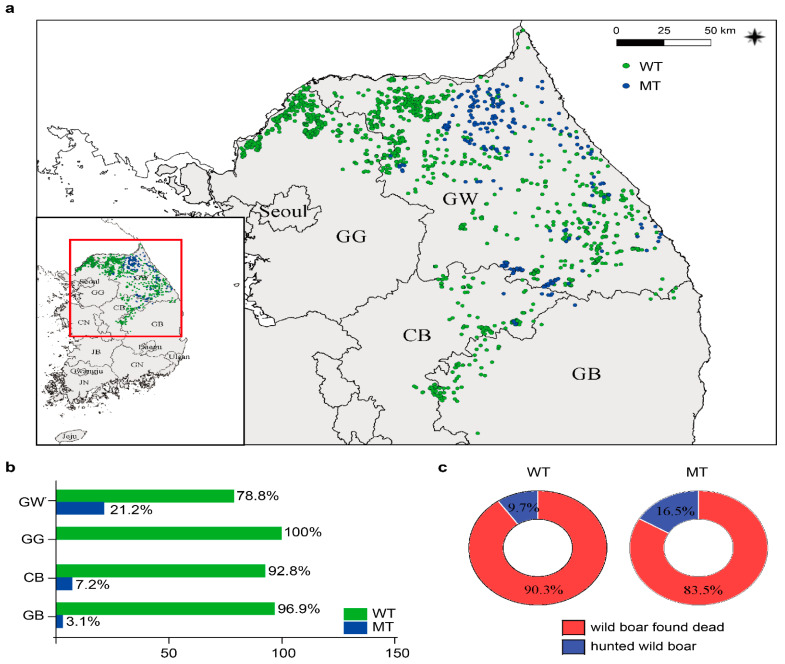
Spatial spread of different ASF strains in Korea from 2019 to 2022. (**a**) The green dots represent wild-type viruses, and the blue ones represent mutant viruses. (**b**) The graph shows the incidence rates of wild and mutant strains by region. (**c**) The wild type is on the left; the mutant-type virus is on the right; the hunted boar is in blue, and the carcass is in red. GW, Gangwon-do; GG, Gyeonggi-do; CB, Chungcheongbuk-d; GB, Gyeongsangbuk-do.

We confirmed the mutation sites in the MGF360-1La and MGF360-4L regions of the Korea/IJ702/2020 strain (Figure 5 and Table 2). We then analyzed ASFV samples from 2019 to 2022 to investigate whether the identified mutations also exist in other ASFV strains. Therefore, we performed partial PCR and sequencing analysis targeting the MGF360-1La and MGF360-4L regions. The mutant virus first discovered in Inje spread to Gangwon-do and moved south to Chungcheongbuk-do in November 2021. The mutant type was most frequently identified in Gangwon-do, followed by Chungcheongbuk-do and Gyeongsangbuk-do, adjacent to Gangwon-do. In contrast, no mutant viruses were identified in Gyeonggi-do (Figure 6a,b). The wild type spread more widely without being confined to specific regions. In contrast, the mutant virus showed a pattern of sporadic occurrence in the western regions.

Furthermore, we compared the occurrence rate of genetic mutations between hunted wild boars and carcasses. The wild type was identified in 90.3% of carcasses and 9.7% of hunted boars. The mutant types were identified in 83.5% of carcasses and 16.5% of hunted boars (Figure 6c). Therefore, approximately 5% more mutant forms were identified in the carcasses compared to the wild type. However, further research is needed to determine if this phenomenon is characteristic of the mutant virus or influenced by other factors.

## 4. Discussion

Partial nucleotide sequencing of specific ASFV regions is traditionally used to diagnose ASF and determine ASFV genotypes [51]. Analyzing ASFV genome information is essential as it broadens our understanding of the circulating ASFV variant characteristics, genetic diversity, and evolutionary pathways [52]. ASF first occurred in Yeoncheon in 2019 and disseminated toward the east in 2020, before progressing southward in 2021 and 2022. We analyzed the ASFV genome to investigate the characteristics of the ongoing wild boar ASF outbreaks. ASFV detected in Korean wild boars belonged to genotype II, remarkably similar to other strains in the genotype II group, particularly the Georgia 2007/1, ASFV-wbBS01, China/2018/AnhuiXCGQ, Pig/HLJ/2018, CAS19-01/2019, and Wuhan2019-1 strains. ASFV genome similarity analysis confirmed that the genetic nucleotide sequences of the Yeoncheon and Hwacheon strains were identical, whereas the sequence of the Inje strain differed slightly from the sequences of these two strains. We also identified SNPs within MGFs 360-1La and 360-4L. These SNPs distinguished the Inje strain from the Yeoncheon, Hwacheon, and Georgia 2007/1 strains. Furthermore, no differences were observed between the *MGF 360-1La* and *360-4L* genes in the ASFV isolates from domestic farms in Korea. These results indicate that certain viral strains, such as the Inje strain, occur only in wild boars. The identification of new variants holds the potential for the strategic management and control of ASF in both wild boar and domestic pig populations. Because it allows for the adjustment of response measures to effectively contain ASFV spread and prevent further outbreaks, continuous efforts in identifying new ASFV variants via genome analysis are emphasized.

ASFV, the only member of the *Asfvirus* genus in the Asfarviridae family, has a high molecular weight and linear double-stranded DNA, with a genome size of 170–193 kb, containing 150–167 ORFs, one-third of which have unknown functions [53,54]. ASFV has a relatively low mutation frequency, attributed to the proofreading activity of DNA polymerase and repair mechanisms of the virus [55]. The primary factor responsible for genome length and gene number differences among viruses is ORF gain or loss in the MGFs [12]. ASFV genomes typically contain a conserved central and variable region at both ends, including five MGFs, and most genome variations are attributed to the varied number of MGF genes in the left and right variable regions [56]. ASFV has five MGFs, namely, MGF-100, -110, -300, -360, and -505, which can modulate the immune response of the host and exhibit host specificity [57]. However, the functions and mechanisms of these MGFs have only been partially studied. According to our results, mutant-type MGF-360 1La and -360 4L were present in 16.5% of hunted wild boars, which is 1.7-fold higher than seen in wild types. More than 80% of the mutant types were found in wild boar carcasses, suggesting that the mutant type does not affect virulence. However, additional molecular studies are needed to understand the basis for the observed 1.7-fold increase in the survival of the mutant types compared with that of the wild type. We obtained limited information from genetic analysis and could not determine whether the identified mutations increased the severity of the disease. In addition, further research is necessary to evaluate the effect of the mutations identified in MGFs 360-1La and 360-4L on the protein structure and the signaling of the host cell immune response. In particular, the emergence of new variants may necessitate the development of new vaccines or updating of existing ones. Understanding the genetic characteristics of these variants can aid in the development of effective vaccines targeted to combat specific mutations.

This study identified a new ASFV variant form for the first time in the Inje region. However, the actual origin of the mutation is unknown and several possibilities are being considered. First, owing to the pattern of domestic ASF outbreaks spreading from the western to the eastern regions, the mutation possibly occurred naturally during this spread or a new type of virus from an external source rather than the domestic virus was introduced. However, extensive research is necessary to elucidate the fundamental origin of the emergence of domestically mutated forms during ASF outbreaks. The transboundary spread of ASF raises concerns regarding variant transmission to diverse regions. The data on identified ASF variants obtained from this study can facilitate in preventing its dissemination via international cooperation and exchange of information.

Genetic epidemiology is essential for determining the origin of a virus, creating maps for prevention, and observing its spread. Therefore, the newly identified mutation in Germany can be used as a genetic marker to investigate the genetic epidemiology of various ASFV occurrences [27]. The mutant type was detected most frequently in Chungcheongbuk-do and Gyeongsangbuk-do after its initial detection in Gangwon-do. During the same period, ASF persisted in Gyeonggi-do; however, the mutant type was not detected. In this study, we were able to confirm the geographically clustered occurrence patterns of ASFV variants based on the genetic characteristics discovered. The newly identified mutation sites can be used as genetic markers to track the transmission pathways of ASFV outbreaks in South Korea. With this information, we can elucidate the transmission pathway of ASFV between wild boars and domestic pigs, and utilize the identified markers to differentiate the spatial and temporal variations in ASFV variants.

In conclusion, during the cyclic domestic occurrence of ASF in 2019–2022, we identified characteristic mutations in the ASFV genome through genetic ASFV analysis in wild boar, specifically in the MGF 360-1La and MGF 360-4L regions. These mutations exhibited a clustered pattern of ASF incidence in the local area. The results can improve our understanding of the spread of ASF through genetic ASFV variant diversity. Without a vaccine against ASFV, the best approach to control and prevent ASFV spread is through monitoring and using the identified characteristic mutations as genetic markers for genetic epidemiological analysis. Furthermore, we will continue to analyze the characteristics of ASFV strains in wild boar populations through genomic analysis to control ASFV infection and aid the development of effective drugs.

## Figures and Tables

**Table 1:** ASFV genome information used in this study.

Strain	Accession Number	Country	Year	Host	Length (bp)	Genotype	Ref.
Korea/YC1/2019	ON075797	Korea	2019	Wild boar	188,950	II	[31]
Korea/HC224/2020	OP618183	Korea	2020	Wild boar	188,645	II	Unpublished
Korea/IJ702/2020	-	Korea	2020	Wild boar	188,598	II	Unpublished
Georgia 2007/1	FR682468.2	Georgia	2007	Pig	190,584	II	[32]
ASFV/pigChina/CAS19-01/2019	MN172368	China	2019	Pig	189,405	II	[33]
Belgium 2018/1	LR536725	Belgium	2018	Wild boar	189,404	II	[34]
ASFV-wbBS01	MK645909	China	2018	Wild boar	189,394	II	Unpublished
Arm/07/CBM/c2	LR812933	Armenia	2007	Pig	190,145	II	[35]
Wuhan2019-1	MN393477	China	2019	Pig	190,576	II	Unpublished
Tanzania/Rukwa/2017/1	LR813622	Tanzania	2007	Pig	183,186	II	[36]
China/2018/AnhuiXCGQ	MK128995	China	2018	Pig	189,393	II	[37]
ASFV/POL/2015/Podlaskie	MH681419	Poland	2015	Pig	189,394	II	[25]
Odintsovo_02/14	KP843857	Russia	2014	Wild boar	189,333	II	[38]
Estonia 2014	LS478113	Estonia	2014	Wild boar	182,446	II	[39]
ASFV_HU_2018	MN715134	China	2018	Pig	190,601	II	[40]
ASFV/Ulyanovsk 19/WB-5699	MW306192	Russia	2019		189,263	II	[41]
Pig/HLJ/2018	MK333180	China	2018	Pig	189,404	II	[42]
Spain-E75	NC_044958.1	Spain			181,187	I	[43]
Portugal-L60	NC_044941	Portugal	1960		182,362	I	[44]
HeN/ZZ-P1/21	MZ945536	China	2021	Pig	171,235	I	[45]
SD/DY-1-21	MZ945537	China	2021	Pig	172,025	I	[45]
72407/Ss/2005	MN270978	Italy	2005	Sus scrofa	181,699	I	Unpublished
Mkuzi/1979	AY261362	South Africa	1979		192,714	I	Unpublished
Liv13/33	MN913970	Zambia	2017	Ornithodoros moubata	188,277	I	[46]
Warmbaths	AY261365	South Africa	1987	Tick	190,773	III	Unpublished
Namibia/Warthog	AY261366	Namibia	1980	Warthog	186,5258	IV	[47]
Ken06.Bus	KM111295	Kenya	2006	Pig	184,368	IX	Unpublished
Uganda/R35	MH025920	Uganda	2015	Pig	188,629	IX	Unpublished
Tengani62	AY261364	Malawi	1962	Pig	185,689	V	Unpublished
MalawiLil-20/1	AY261361	Malawi	1983	Tick	187,162	VIII	[48]
ASFV Ken.rie1	LR899131	Kenya	2019	Tick	190,592	X	Unpublished
Kenya1950	AY261360	Kenya	1950	Pig	193,886	X	[49]
BUR/18/Rutana	MW856067	Burundi	2018	Pig	176,564	X	[50]

**Table 2:** Genetic differences in Korean ASFV strains compared with other ASFV strains.

Name	Accession Number	Nonsynonymous		Synonymous
		MGF 360-1La	MGF360-4L	MGF 360-10L
Korea/YC1/2019	ON075797	A	C	G
Korea/HC224/2020	OP618183	A	C	G
Korea/IJ702/2020	-	G	G	C
Georgia2007	FR682468	A	C	C
HU2018	MN715134	A	C	C
Pig/HLJ	MK333180	A	C	C
AnhuiXCGQ/2018	MK128995	A	C	C
wbBS01	MK645909	A	C	C
Pig/china2019	MN172368	A	C	C
Wuhan2019	MN393477	A	C	C
Odintsovo	KP843857	A	C	C
ulyanovsk19	MW306192.1	A	C	C
Arm/07/CBM	LR812933	A	C	C
Belgium2018	LR536725	A	C	C
Tanzania2017	LR813622	A	C	C
Podlaskie2015	MH681419	A	C	C

## Data Availability

Not applicable.
